# Measurement properties of the Swedish clinical outcomes in routine evaluation outcome measures (CORE-OM): Rasch analysis and short version for depressed and anxious out-patients in a multicultural area

**DOI:** 10.1186/s12955-022-01937-7

**Published:** 2022-02-19

**Authors:** Louise Danielsson, Magnus L. Elfström, Javier Galan Henche, Jeanette Melin

**Affiliations:** 1 Institute of Neuroscience and Physiology, Department of Health and Rehabilitation, Sahlgrenska Academy, University of Gothenburg, Box 455, 405 30 Gothenburg, Sweden; 2 Angered Hospital, Box 63, 422 24 Gothenburg, Sweden; 3 Division of Psychology, School of Health, Care and Social Welfare, Mälardalen University, Eskilstuna, Sweden; 4 RISE Metrology, Research Institutes of Sweden, Gothenburg, Sweden

**Keywords:** Psychometrics, Internal validity, Patient-reported outcome measures, Item response theory

## Abstract

**Introduction:**

The Swedish version of the patient-reported Clinical Outcomes in Routine Evaluation Outcome Measures (CORE-OM) has demonstrated high reliability and acceptable convergent validity in explanatory factor analyses. However, the fundamental scale properties have not yet been validated according to item response theory. The aim of this study was to analyze the measurement properties of the Swedish CORE-OM in a cohort of psychiatric out-patients with depression and anxiety in a multicultural area and to explore combinations of items based on shorter versions of the scale (CORE-10, CORE-6D) to improve measurement properties.

**Methods:**

Data from CORE-OM assessments of 337 patients were analyzed using Rasch analysis. The patients had a mean age of 30 ± 14 years, the majority were women (72%). Requirements for measurement properties were checked: overall model fit, item fit residuals, targeting, internal consistency, differential item functioning and thresholds. Sensitivity to change was also analyzed.

**Results:**

The CORE-OM showed high internal consistency (person separation index = 0.947) and adequate targeting, but there was overall model misfit (item trait interaction χ^2^ = 917.53, *p* < 0.001), indication of local dependency, and differential item functioning in 9 items. The risk items showed problems with disordered thresholds. The emotional component of the shorter CORE-6D showed the best fit for our sample. Adding 3 items to include depressive and trauma-related content resulted in a unidimensional 8-item set with acceptable reliability, model fit, targeting and sensitivity to change.

**Conclusion:**

For out-patients with diagnosed depression or anxiety in a multicultural area, the Swedish CORE-OM showed high internal consistency, but also validity problems. Based on the shorter CORE-6D version, a unidimensional 8-item set could be an alternative brief measure of psychological distress for this population, but further validity studies are required. Qualitative studies exploring the CORE-OM items in non-native speakers are also warranted.

## Introduction

Patient reported outcome measures (PROMs) are defined as a report of the status of a patient’s health condition that comes directly from the patient without interpretation from a clinician or anyone else [1]. PROMs are being increasingly used in mental health to capture physical, psychological and social aspects of the patient’s health and wellbeing [2]. While the systematic use of PROMs facilitates communication and shared decision-making between patient and health care provider, there are practical and sociocultural considerations to routine use [2]. Moreover, the measurement needs to be incorporated so that it does not misdirect the focus of the clinical encounter or become a burden to patients or health care professionals [2].

People who seek health care because of psychological distress commonly present symptoms of depression, anxiety, or somatization. For these patients, counting for approximately 50% of primary health care visits [3], it is essential that PROMs are psychometrically sound, relevant to their problems and sensitive to monitor treatment progress [4]. Many well-established psychiatric scales are symptom-based and target specific diagnoses while measuring depression and anxiety as two separate constructs, such as the Beck Depression and Anxiety Inventories [5, 6], and the Hospital Anxiety and Depression Scale [7]. Since comorbidity between depression and anxiety is high [3], and less specific problems such as fatigue and somatization are common [8, 9], relevant PROMs need to capture a broad panorama of distress in the depressed and anxious population.

The Clinical Outcomes in Routine Evaluation – Outcome Measure (CORE-OM) was developed in the United Kingdom as a generic self-report measure of psychological distress, primarily to evaluate psychological treatment in clinical practice [10]. The scale covers core characteristics of psychological distress, based on what patients commonly present to clinicians, and is not restricted to a specific psychiatric diagnosis [10–12]. The CORE-OM comprises four conceptual domains measuring *problems/symptoms, life/ social functioning, subjective well-being and risk to self or others.* The domain scores are to be explored only where particularly indicated clinically or for specific research interest. The scale is free to use for non-commercial purposes.

A qualitative study found that patients perceived the CORE-OM clear, understandable and useful [13]. The questions increased their self-awareness and made them reflect on their present and future situation [13]. While patients seem to regard the CORE-OM a valuable tool during treatment, the purpose of assessment needs to be clearly communicated [14].

According to principles of Classical Test Theory (CTT), the original English CORE-OM has high internal and test–retest reliability, good sensitivity to change and good convergent validity [10]. It has been translated into 54 languages and dialects [15]. Most psychometric studies have been conducted in primary health care settings with depressed or anxious adult patients, but the scale has also been validated in adolescents [16] and in people with learning disabilities [17], eating disorders [18], tinnitus [19] and substance misuse [20]. A shorter 10-item version has shown high acceptability in terms of readability, high reliability and high convergent validity with the full CORE-OM [21]. Recently, a preference-based index with 6 items, CORE-6D [22, 23]. was developed from the CORE-OM.

In a study that compared 21 CORE-OM studies conducted in different countries [15], the different translations of the CORE‐OM in samples with mainly native speakers showed comparable results. Internal consistency and convergent validity are high but there are recurrent problems with floor effects and low test–retest stability for the *risk to self or others* domain [15]. None of the validity studies applying factor analysis has been able to replicate the intended four-factor structure of the CORE-OM, suggesting an area for modification of the scale [15]. While one study has suggested either a one‐factorial or a two‐factorial structure for their data, most studies suggest a latent structure of three major components: a positively formulated dimension measuring strengths, a negatively formulated dimension measuring weaknesses, and a dimension consisting of the risk items.

Few studies evaluating the CORE-OM have addressed the fundamental measurement properties of the scale using the framework of item response theory (IRT), such as Rasch analysis. This framework is useful to study dimensionality and item functioning of a scale to understand and optimize valid and reliable measures. Recently, the Rasch methodology was used in a psychometric study of the Russian translation of CORE-OM [24], indicating a need for further research on dimensionality and potential item bias for gender and diagnostic groups. Mavranezouli et al. [22, 23] used Rasch analysis to derive items to the CORE-6D, generating a 2-dimensional (emotional and physical components) health index that showed good model fit, no item bias and acceptable reliability.

The Swedish version of CORE-OM has been validated according to procedures in line with CTT, using explanatory factor analysis, demonstrating high reliability and acceptable convergent validity [25]. However, to the best of our knowledge, the fundamental scale properties of the Swedish CORE-OM have not yet been analyzed using IRT. Furthermore, in the initial validation study of the Swedish version it was recommended to examine the version in more diverse samples [25].

Scales that are intended to provide outcome measures do not only have to provide valid and reliable measurement results, changes in health status also need to be accurate. Thus, a scale such as CORE-OM is also subject to evaluation of sensitivity to change. Based on group level analyses, both the original UK version [10] and the Swedish version [25] of CORE-OM has shown good sensitivity to change. Those studies did not, however, investigate item stability over time, which is a key to comparability and can be analyzed using item response theory [26–28]. For clinical practice, it is important to be able to measure individual patient’s change during the treatment, and it is therefore warranted to assess sensitivity to change on an individual level accounting for the measurement uncertainties for the individual measure [26].

The aim of this study was to analyze the measurement properties of the Swedish CORE-OM in a cohort of psychiatric out-patients with depression and anxiety in a multicultural area and to explore combinations of items based on shorter versions of the scale (CORE-10, CORE-6D) to improve measurement properties.

## Methods

This psychometric study used modern test theory, and, more specifically, Rasch analysis. Rasch analysis, increasingly used in health research on patient-reported outcomes [29], offers a method of investigating whether the required measurement properties of a scale are supported or not, to guide whether arithmetic operations can be undertaken. The Rasch model is a unidimensional model with two main assertions, namely, that: a) the easier an item, the more likely it will be affirmed; and b) the “more” of the attribute a patient has, the more likely they will affirm an item [30]. For a scale where a sum score is calculated, such as the CORE-OM, these assumptions should underpin the scale construction. Exploring these basic properties of a scale is essential to ensure that data can be regarded as interval data, which is required for calculating sum scores and changes and to perform parametric statistical tests.

The Swedish CORE-OM [25] consists of 34 items consisting of statements which the patient responds to on a 5-grade scale: 0 = not at all, 1 = only occasionally, 2 = sometimes, 3 = often, and 4 = most or all the time. Eight items are inversely worded (in all domains) and consequently rescored in the Rasch analysis. The items represent four domains: life/social functioning (12 Items), problems/symptoms [12 items], risk to self or others [6 items] and subjective wellbeing [4 items]. The shorter CORE-10 consists of ten statements derived from the 34 items: life/social functioning [3 items], problems/symptoms [5 items] and risk [1 item].

In the CTT psychometric study, internal consistency of the full 34 item version of the Swedish CORE-OM was 0.93 in a non-clinical sample and 0.94 in a clinical sample [25]. Test–retest reliability, using intraclass correlation, was between 0.78–0.80 for the different domains. Excluding the six *risk* items from the total CORE-OM, test–retest stability was 0.83 [25].

### Participants and study setting

We included adults experiencing psychological problems (depressive-, anxiety- or trauma-related), who had been referred or self-referred to out-patient psychiatric care. Exclusion criteria were substance abuse or psychotic disorders. Participants were recruited from a mental health clinic in a metropolitan area in Sweden, between January 2017 and September 2020. Included in the study during this time frame were 337 consecutive out-patients with diagnosed depression or anxiety. The clinic, organized at a level between primary health care and specialized psychiatry, has a multi-professional psychiatric team, offering medical, psychological and rehabilitation interventions. The clinic is situated in a district where the inhabitants have lower socioeconomical resources than the region average and around 50% are born abroad [31]. The clinic gives high priority to young adults seeking help for psychological problems. Patients are generally in treatment for 6–12 months. Ethical approval for the study was obtained from the Swedish Ethical Review Authority, reference number 2020–04,181.

### Data generation and power considerations

**Fig. 1:**
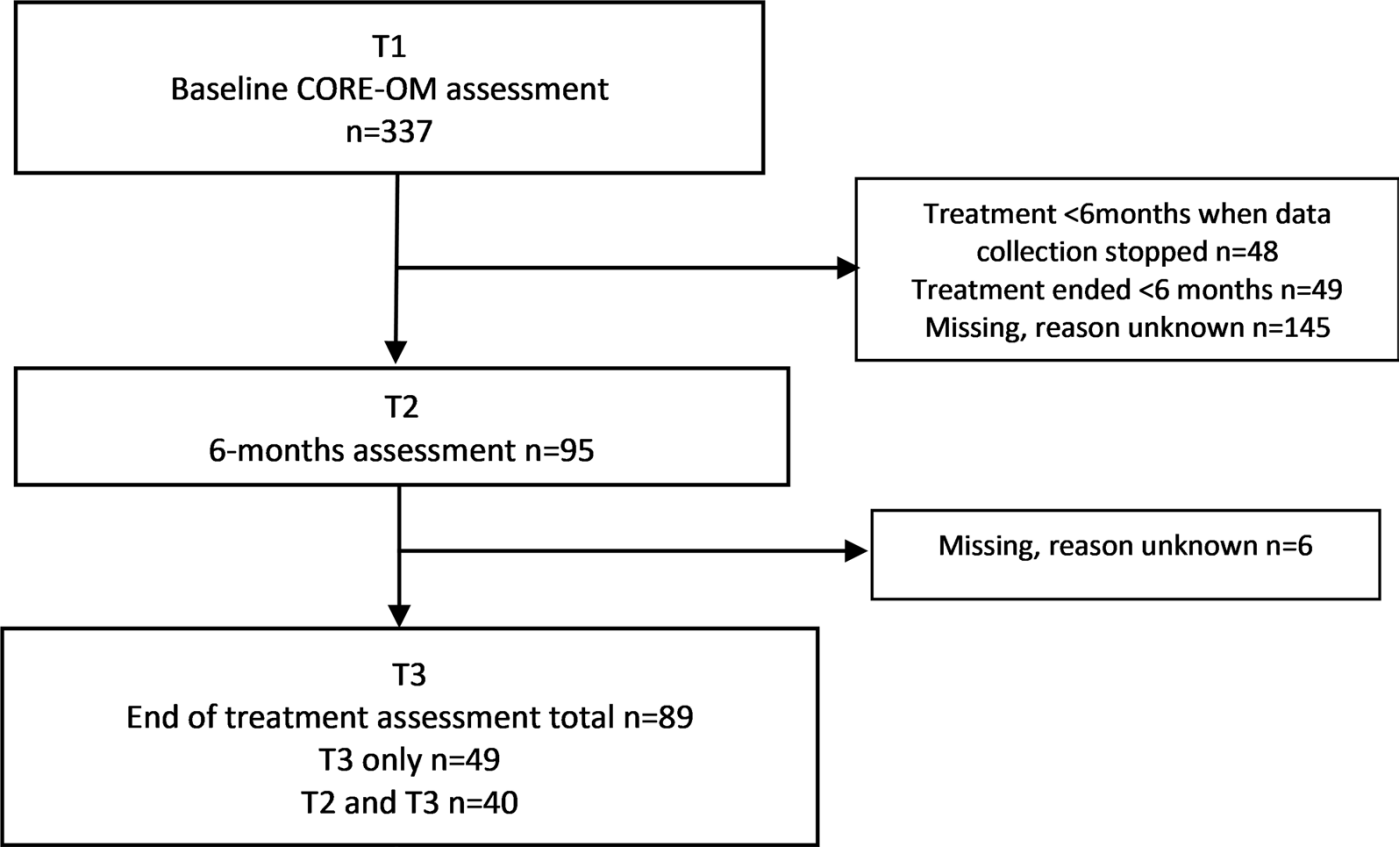
Flow chart of the participants at different time points during the study

Participants were routinely assessed with CORE-OM at their first visit, and those who continued with treatment were then followed up at 6 months, and/or at the end of treatment. In this naturalistic study, duration of treatment differed and, thus, the timepoint for the end of treatment differed. End of treatment sometimes occurred before 6 months resulting in a lack of 6 months assessment for these participants. Also, some participants had not yet been in treatment for 6 months when data collection ended. The flow of study participants related to data collection at different time-points are visualized in Fig. 1.

All participants filled in the CORE-OM in the format of a paper-pen questionnaire which was distributed to them by staff at the clinic either in the waiting room or during the appointment. The health care professional who collected the questionnaire was able to assist should the participant have questions or problems to understand an item. They were reassessed in the same way at follow-ups.

In general, when the person-to-scale distribution is well targeted, more items improve reliability of person measures, and vice versa, more persons improve reliability of item measures [32]. A larger sample size is preferable in scales with several response categories, such as the CORE-OM. While more persons are preferable, sample sizes > 500 increase the risk of Type I errors and, thus, sample sizes between *N* = 250 to *N* = 500 may provide a good balance [33], which guided the sample size for this study. Pooling data from repeated measures has been recommended as an option to increase the sample size for Rasch analyses for more stable calibrations [34, 35], and enable assessments of item stability across different timepoints to ensure comparability [35] as well as assessing sensitivity to change in person measures [26, 28].

### Data analysis

Data was initially recorded and managed in Excel. The software RUMM2030 was used for the psychometric analyses. In the Rasch analysis, statistics were calculated and interpreted checking requirements for measurement properties while considering the qualitative meaning of the items in an iterative process.

- *Person reliability*. Reliability of person measures was analyzed using the person separation index (PSI), a reliability statistic that is interpreted in the same way as Cronbach’s alpha, suggesting that a minimum PSI value of 0.7 is required for group-decisions and 0.85 for use at the individual level [36].
- *Model fit: item-trait interaction and individual item fit*. Essentially, Rasch analysis looks at the deviation of the observed data from the model expectation. Both the overall model fit, and the individual item fit are analyzed. Results are reported as a series of chi-square statistics (both for item-trait interaction and for individual items) and fit residuals demonstrating the discrepancy between expected and observed data. Where an item fits the model, the chi-square (χ^2^) probability is non-significant (Bonferroni corrected p-value) and fit residuals should be within a desirable range of ± 2.5 [37].
- *Person-item threshold distribution.* The balance between person and items are checked for and visualized by the person-item threshold histogram, which shows the targeting of the scale, that is, if the items capture the subjects under study.
- *Differential item functioning (DIF)* was analyzed to check if items worked in the same way across groups of patients of different gender (men/women), age (young adults 18–27 years/adults > 27 years), and timepoints T1-T3 (baseline, after 6 months treatment, end of treatment).
- *Local dependency*. Local independence means that the entire correlation between the items should be captured by the underlying construct (i.e. the latent trait, here, psychological distress). Should there be correlation values above a relative cut off greater than 0.20 above the average correlations [38], this would indicate local dependency (that a response to one given item is not independent from the response to another item).
- *Threshold ordering*. With polytomous scales, the response options should work so that the transition from one category to the next follow the underlying trait. That means that as the trait (psychological distress) increases, so does the response option. When an item does not follow this assumption, the Rasch analysis indicates disordered thresholds. Ideally, all thresholds should be significantly different from each and reflect an increase in psychological distress.
- *Unidimensionality.* The Rasch model assumes a single, unidimensional construct, which is a prerequisite to adding items into a sum score. Unidimensionality means that a single construct (e.g. psychological distress in people with depression and anxiety disorders) is being measured by a set of items. In a principal component analysis, two subsets of items with the highest and lowest loadings were created. The person estimates from these two subsets of items were subjected to a series of t-tests. A non-significant difference between the two person estimates would support the unidimensionality of the scale. The percentage of tests outside the -1.96 to 1.96 range should not exceed 5% [36].

To investigate the sensitivity to change, *t tests* were computed for individuals as well as group level. For individual tests the number of significant changes were computation followed recommendations by Anselmi et al. [26];${t}_{j}=\left({\theta }_{j1}-{\theta }_{j2}\right)/\sqrt{{SE}_{j1}^{2}+{SE}_{j2}^{2}}$, were ${\theta }_{j2}$ and ${\theta }_{j1}$ are the individual person estimates for two time points. Comparisons were done both between for *Time 1* (baseline) with *Time 2* [6 months] and for *Time 1* (baseline) with *Time 3* (end of treatment). For group comparisons, both intention-to-treat (ITT) and per protocol (PP) analyses were computed and Cohen’s d were calculated as mean differences divided by the pooled standard deviation were interpreted as 0.2 and < 0.5, small effect; 0.5 and < 0.8, moderate effect; and ≥ 0.8, large effect [39].

## Results

Three hundred and thirty-seven patients were included, see Table 1. The mean age of the participants was 33.1 years, SD 14.0 years. Of the 337 participants, 27% were men, 72% were women and 1% did not define their gender. Ninety-five of the participants had a second assessment at 6 months and 89 of the participants had an end of treatment assessment (Table 1 and Fig. 1). Among the many participants who lacked data from the second and third assessment we found no significant difference compared to the whole sample in terms of their age (mean 35.8 years), gender (75% female) or symptom severity (CORE-OM mean = 2.0). For more stable calibrations, available data from the three assessments were included in the Rasch analysis.

**Table 1:** Participant characteristics and CORE-OM scores at different points of time

		n = 337		
Age group	18–27 years	167 (49.6%)		
	> 28 years	170 (50.4%)		
Gender^1^	Men	91 (27%)		
	Women	243 (72%)		
Main diagnosis^2^	Depressive disorder (F32-33)	20/109 (18%)		
	Anxiety disorder (F41-42)	53/109 (49%)		
	Post-traumatic stress disorder or reactions to severe stress (F43)	28/109 (26%)		
	Other main psychiatric diagnosis^3^	8/109 (7%)		
		Baseline (T1) n = 337	6-months (T2) n = 95	Treatment end (T3) n = 89
CORE-OM score	Mean score 0–4 (SD)^4^	2.15 (0.61)	1.83 (0.64)	1.22 (0.68)

^1^Other gender are not presented in the table due to very small numbers

^2^Data on diagnosis were only available for n = 109 individuals

^3^Mainly personality or neuropsychiatric disorders

^4^Higher score indicate more psychological distress

### ***Measurement properties of the CORE-OM ***[34 items]

*Person reliability.* The initial Rasch analysis showed a person separation index of 0.947, which indicates high reliability.

*Model fit.* Item trait interaction for the whole scale showed a significant χ^2^ probability (*p* < 0.001). Item fit statistics (Table 2) corroborated the deviation from the model, displaying fit residuals outside the desired range of ± 2.5 with significant p-values in 9 items: 1, 3, 8, 11, 17, 19, 23, 27, 31. These items were from the *wellbeing* (n = 2), *function* (n = 3) and *problem* (n = 4) domains. Three of the items were positively worded and six were negatively worded.

**Table 2:** Fit statistics of the Swedish CORE-OM with items ordered from low to high location (easy to difficult items)

Item	Item descriptor	Domain	Location	SE	FitRes	Probability	Thresholds
**2**	I have felt tense, anxious or nervous	P	− 1.218	0.054	− *2.65*	0.0325	Organized
20	My problems have been impossible to put to one side	P	− 0.942	0.048	− 2.28	0.0024	Organized
13	I have been disturbed by unwanted thoughts and feelings	P	− 0.88	0.050	− 0.99	0.1124	Organized
5	I have been totally lacking in energy and enthusiasm	P	− 0.684	0.048	− *2.86*	0.0004	Organized
4	I have felt OK about myself	W	− 0.581	0.051	0.451	0.6372	Organized
**23**	I have felt despairing or hopeless	P	− 0.581	0.047	− *6.67*	0.000*	Organized
**27**	I have felt unhappy	P	− 0.57	0.047	− *5.9*	0.000*	Organized
**18**	I have had difficulty getting to sleep or staying asleep	P	− 0.514	0.041	*4.044*	0.00035	Organized
30	I have thought I am to blame for my problems and difficulties	P	− 0.486	0.044	*4.212*	0.0581	Organized
**28**	Unwanted images or memories have been distressing me	P	− 0.482	0.045	− 0.11	0.0429	Organized
14	I have felt like crying	W	− 0.452	0.048	− 0.24	0.2855	Organized
17	I have felt overwhelmed by my problems	W	− 0.425	0.044	− *6.48*	< 0.001*	Organized
11	Tension and anxiety have prevented me doing important things	P	− 0.391	0.044	− *3.92*	< 0.001*	Organized
8	I have been troubled by aches, pains, or other physical problems	P	− 0.354	0.042	*7.191*	< 0.001*	Disorganized
31	I have felt optimistic about my future	W	− 0.331	0.045	*8.722*	< 0.001*	Disorganized
1	I have felt terribly alone and isolated	F	− 0.308	0.046	− *4.12*	< 0.001*	Organized
**10**	Talking to people has felt too much to me	F	− 0.272	0.046	− 1.18	0.1382	Organized
32	I have achieved the things I wanted to	F	− 0.23	0.05	0.406	0.2999	Organized
12	I have been happy with the things I have done	F	− 0.13	0.052	0.298	0.9485	Organized
**7**	I have felt able to cope when things go wrong	F	− 0.057	0.05	*2.884*	0.0249	Organized
25	I have felt criticized by other people	F	0.008	0.045	*2.671*	0.0014	Organized
29	I have been irritable when with other people	F	0.115	0.046	*3.562*	0.0007	Organized
**3**	I have felt I have someone to turn to for support when needed	F	0.152	0.043	*6.356*	< 0.001*	Organized
26	I have thought have no friends	F	0.153	0.042	*2.493*	0.0156	Organized
**15**	I have felt panic or terror	P	0.16	0.045	− 1.21	0.0252	Organized
19	I have felt warmth or affection for someone	F	0.237	0.043	*9.894*	< 0.001*	Organized
21	I have been able to do most things I needed to	F	0.326	0.049	− 0.33	0.7865	Organized
24	I have thought it would be better if I were dead	R	0.468	0.042	− *3.09*	0.0057	Organized
33	I have felt humiliated or shamed by other people	F	0.769	0.046	1.64	0.3683	Organized
9	I have thought of hurting myself	R	0.923	0.046	0.829	0.3650	Disorganized
**16**	I made plans to end my life	R	1.4	0.053	− 2.47	0.0578	Disorganized
34	I have hurt myself physically or taken dangerous risks with my health	R	1.426	0.055	1.059	0.1568	Disorganized
22	I have threatened or intimidated another person	R	1.804	0.066	1.244	0.0121	Disorganized
6	I have been physically violent to others	R	1.945	0.086	0.523	0.0784	Disorganized

Bold item numbers = items used in the CORE-10. P = Problems/symptoms, W = Well-being, F = Life/Social functioning, R = Risk of harm to self or others. FitRes = Fit residuals (in italics when outside desired range ± 2,5 and p-value (marked with an asterisk when significant)

**Fig. 2:**
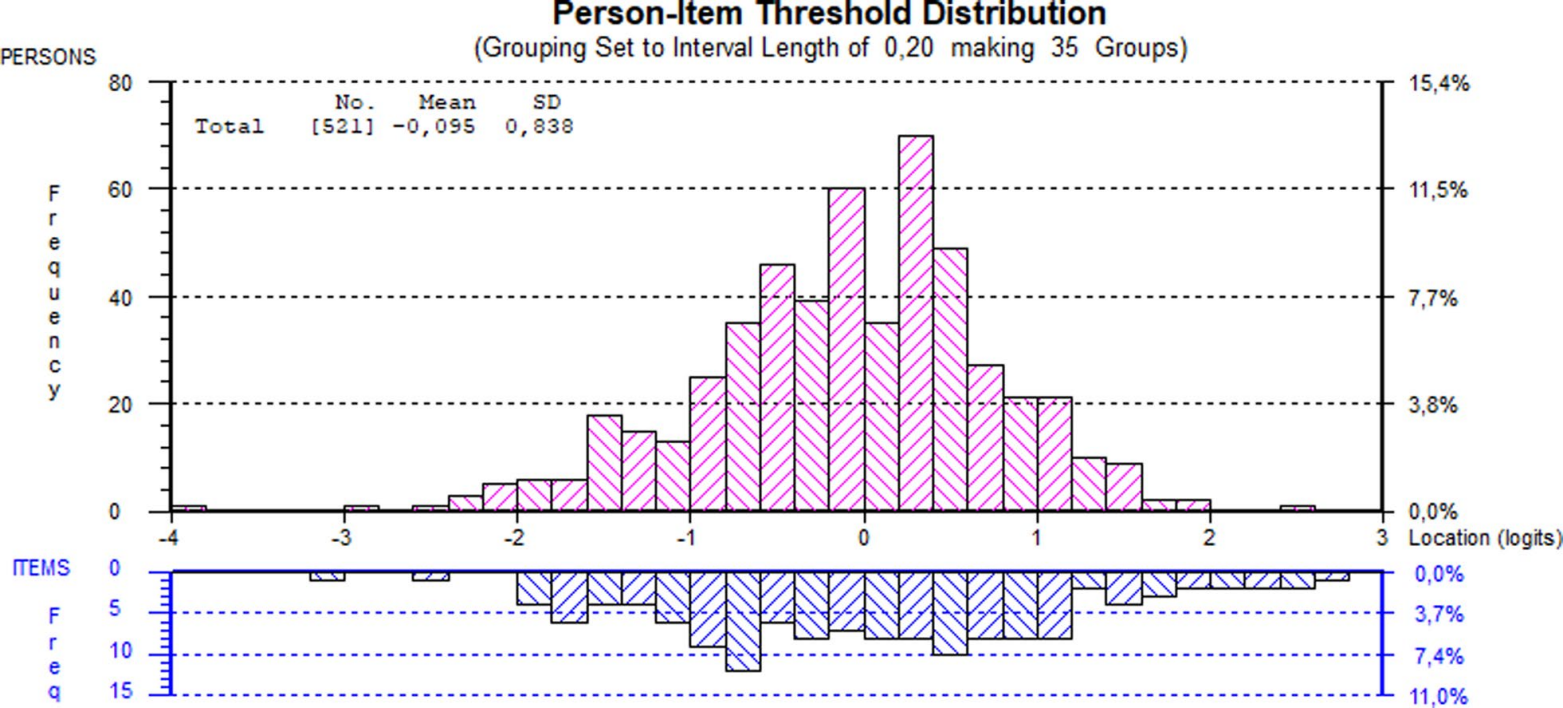
Targeting of the Swedish CORE-OM in a cohort of depressed and anxious patients

*The person-item threshold distribution* showed balance between persons and items, see Fig. 2. The mean logit of persons was -0.096, SD 0.84, which means a well targeted scale (ideal mean value with a perfect normal distribution is 0). Figure 2 also shows that, while the scale items capture an adequate range of distress (-3 to 3 logits), there were some items with high difficulty (higher positive logit values) that very few or none of the participants affirmed. The highest difficulty items were number 6, 22, 34, 16 and 9 (Table 2), which all belong to the risk domain and represent high risk behavior, such as suicide ideation or violence. Moreover, items from the *problem* and *wellbeing* domains were easiest, while most items from the *function* domain were in the middle of the psychological distress continuum.

*Differential item functioning.* The analysis showed uniform DIF for gender in items 14 (*I have felt like crying*) and 19 (*I have felt warmth or affection for someone*) and DIF for age in items 8 (*I have been troubled by aches, pains or other physical problems)* and 9 (*I have thought of hurting myself*). This means that for these items, given the same level of distress, patients of different gender or age responded differently on those items. Furthermore, five items [2, 8, 19, 23 and 27] showed significant DIF with regards to timepoints, thus, lack of item stability over time.

*Local dependency.* The analysis showed 27 of 561 residual correlations above the average, which suggests local dependency. Among the highest correlations, we found that five correlations were pairs of items from the risk domain, which suggests that they are linked based on similar qualitative meaning. Likewise, other correlations showed item pairs with similar meaning. For example, the correlation between item 21 and 25 could be explained by similarly phrased positive statements, both belonging to the function domain. Items 25 and 33 both belong to the function domain, the subdomain of social functioning. While the 17–23 correlation could not be explained by belonging to the same domain, both items are closely related to what the depressed patient may experience.

*Threshold ordering* was disordered for 7 items (Table 2); 6, 8, 9, 16, 22, 31 and 34, most of which belong to the *risk* domain. A high proportion (49–89%) of respondents rated *not at all* on those items. *Unidimensionality* could not be supported by the *t-test*; 20.9% were outside the desired ± 1.96.

*Sensitivity to change*. 38 of 96 (40%) patients showed a significant improvement between Time 1 (baseline) and Time 2 (after six months), and 61 of 88 (69%) patients showed a significant improvement between Time 1 (baseline) and Time 3 (end of treatment) (*p* < 0.05). Ten patients showed significant deterioration between Time 1 (baseline) and Time 2 (after six months) and three patients between Time 1 (baseline) and Time 3 (end of treatment) (*p* < 0.05). All group comparisons were significant across timepoints for PP and ITT analyzes (*p* < 0.01). Effect sizes were small for all ITT analyses (Time 1-Time 2 d = 0.29; Time1-Time3 d = 0.20) and PP analyses between Time 1 and 2 (d = 0.41), while PP analyses between Time 1 and Time 3 yielded large effect (d = 1.22).

### Analysis of item combinations based on shorter versions of the scale

The item sets previously derived from the CORE-OM to form shorter versions of the scale, were also subjected to Rasch analysis using our data. We also analyzed the CORE-OM without the risk domain. Table 3 shows how the different versions compare regarding overall fit statistics, item bias and threshold ordering. Overall, the reliability of the shorter scales remained high or acceptable (> 0.70). However, only the CORE-6D (emotional component) showed acceptable model fit (p = 0.0113) with no individual item misfit and a unidimensional construct (Table 3).

**Table 3:** Measurement properties of the Swedish CORE-OM, and alternative item sets, in a cohort of depressed and anxious patients

Rasch statistic	Investigated properties/Statistical question	Fit criteria and interpretation	CORE-OM [34 items]	CORE-OM non risk [28 items]	CORE-10 [10 items]	CORE-6D emotional [5 items]	CORE-6D added [8 items]
Person separation index	Are item responses consistent across respondents?	Values > 0.8 individual use, > 0.7 group use	0.95	0.94	0.86	0.73	0.85
Overall model fit, mean (SD)	Do observed items responses correlate with expected responses from the Rasch model?	Perfect fit = mean of 0 and SD of 1 Acceptable fit = SD < 1.5	0.41 (4.00)	0.63 (4.44)	0.20 (3.69)	− 0.10 (1.49)	− 0.02 (1.41)
Item-trait interaction, chi-square p-value	Probability that the overall responses fit the model?	Non-significant Bonferroni-adjusted p-value	917.53 * p* < 0.001	839.78 * p* < 0.001	278.23 * p* < 0.001	56.78 p = 0.0113*	76.11 p = 0.038*
Individual item fit	Do the observed individual item responses correlate with expected responses from the Rasch model?	Fit residual = + / − 2.5 Chi-square p-values nonsignificant (Bonferroni-adjusted). Visual check of item characteristic curves	9 items show misfit and sign p-value	9 items show misfit and sign p-value	5 item show misfit and sign p-value	No item misfit	No item misfit
Differential item functioning	Does any item deviate from the requirement of invariance across groups for gender, age and diagnosis?	Nonsignificant Bonferroni-adjusted probability value	DIF gender items 4, 19; age items 8, 9; time items 2, 8, 19, 23, 27	DIF gender items12, 16; DIF age items7, 16	DIF age item 6 DIF time items 1, 8	DIF age items 5, 16	DIF age item 5
Local dependency	Does any item show dependency on a response to another item?	No correlations above the relative cut off greater than 0.20 above the average correlations	27 (of 561) correlation (relative cut-off 0.17)	24 (of 351) correlations (relative cutoff 0.17)	26 (of 45) correlation (relative cutoff 0.1)	None	None
Thresholds	Do the response categories work as intended, or are there disordered thresholds?	Ordered thresholds	7 disordered thresholds, items 6, 8, 9, 16, 22, 31, 34	1 disordered threshold, item 8	1 disordered threshold, item 16	1 disordered threshold, item 16	1 disordered threshold, item 16
Unidimensionality	Does the questionnaire measure one single dimension?	The proportion of t tests reaching significance should not exceed 5% in the independent t test protocol	20.9%	21.9%	7.29%	1.15%	4.61%

^*^The Bonferroni-adjusted alpha level (0.05/number of items) was 0.01 for CORE-6D and 0.00625 for CORE-6D added

Based on the observations of four different CORE scales, the CORE-6D showed the best model fit for our data. However, since person separation index was not sufficiently high for individual use (0.733), we considered how the item set could be improved. To match our sample, we considered it essential to include items capturing trauma-related and depressive problems. Based on previous statistical analyzes and discussions about the meaning of items we added item 5 (*I have felt totally lacking in energy and enthusiasm*), to account for depressive content, item 13 (*I have been disturbed by unwanted thoughts and feelings*) and item 28 (*Distressing images or memories have been distressing me*), to account for trauma-related content. One of the items, *I made plans to take my life*, however, showed disordered thresholds, but aiming to remain as close as possible to the validated CORE-6D, this item was kept. This item set of 8 items showed satisfactory measurement properties, good reliability for individual use (PSI = 0.85), no local dependency and satisfactory unidimensionality, see Table 3.

**Fig. 3:**
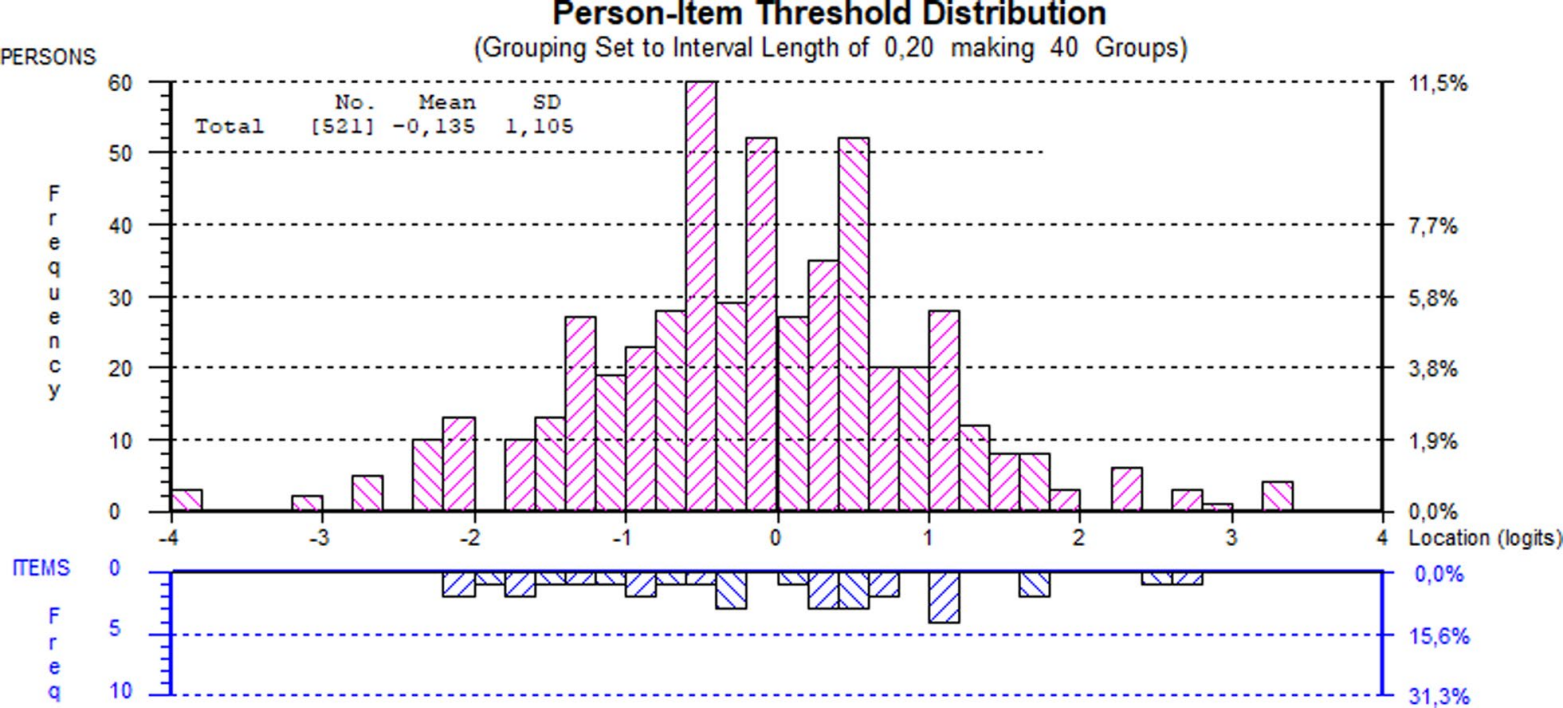
Targeting of the modified CORE-6D (emotional dimension) with 3 added items

Figure 3 shows the person-item threshold distribution and Table 4 provides item fit statistics for the 8 items. They are ordered in a qualitatively sound hierarchy from the easiest to the most challenging item. This means that it is more common to affirm the items on the lower end of the scale, such as having unwanted thoughts and feelings and lacking energy indicating less distress. In contrast, the items on the upper end is more seldom experienced, such as having plans to end life, indicating more severe distress.

**Table 4:** Fit statistics of the modified CORE-6D with 3 added items

Item	Item descriptor	Domain	Location	SE	Fit residuals	*P* value	Thresholds
13	I have been disturbed by unwanted thoughts and feelings	P	− 0.997	0.052	0.728	0.824	Ordered
5	I have felt totally lacking in energy and enthusiasm	P	− 0.790	0.050	− 0.354	0.526	Ordered
28	Unwanted images and memories have been distressing me	P	− 0.591	0.048	− 0.471	0.649	Ordered
1	I have felt terribly alone and isolated	P	− 0.381	0.049	− 2.108	0.048	Ordered
15	I have felt panic or terror	P	0.136	0.049	− 0.788	0.007	Ordered
21	I have been able to do most things I needed to	F	0.309	0.051	2.439	0.535	Ordered
33	I have felt humiliated or shamed by other people	F	0.833	0.050	1.881	1.118	Ordered
16	I made plans to end my life	R	1.482	0.056	− 0.578	0.170	Disordered

P = Problems/symptoms, F = Life/Social functioning, R = Risk of harm to self or others

**Fig. 4:**
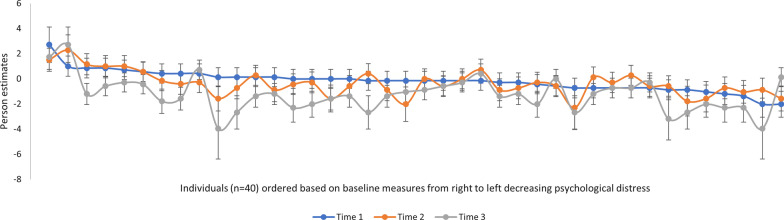
Person estimates for patients with measures from all three time points, ordered according to their baseline measure from right to left decreasing psychological distress. Error bars corresponds to 2SE

*Sensitivity to change.* For this 8-item version of CORE-OM, 1 of 95 (1%) patients showed a significant improvement between Time 1 (baseline) and Time 2 (after six months), and 51 of 88 (58%) patients showed a significant improvement and two patients a significant deterioration between Time 1 (baseline) and Time 3 (end of treatment) (*p* < 0.05). All group comparisons were significant across timepoints, but some effect sizes remained small: PP analyzes of Time 1 and Time 2 (p = 0.020, d = 0.34), Time 1 and Time 3 (*p* < 0.001; d = 1.05), ITT analyzes Time 1 and Time 2 (*p* < 0.001, d = 0.25) and Time 1 and Time 3 (*p* < 0.001; d = 0.44). Moreover, 40 patients had measures from all three time-points. This is illustrated in Fig. 4, showing more measurable changes between Time 1 and 3 than between Time 1 and 2 or Time 1 and 3.

## Discussion

In this study, the Swedish CORE-OM showed high internal consistency, but also several shortcomings in terms of validity and deviation from the expected model. Similar to our findings, reliability was also high in the previous CCT based validity study [25]. In clinical practice, this means that the scale is consistent across respondents in people seeking help for depressive-, anxious or trauma-related problems.

The indicated poor fit to model means that the response pattern to several items were different than what would be expected at a given level of distress. This can partly be interpreted as an unsatisfactory match between the group of persons (here Swedish depressed and anxious out-patients in a multicultural area) and the set of items (the full version of CORE-OM) [40]. In particular, the risk domain showed problems with misfit and disorganized thresholds. In line with our results, Zeldovich et al. [24] also found problems with items displaying high misfit and residuals outside the desired range. Likewise, psychometric problems with the risk domain have been highlighted in other studies [15, 24], in which the authors discuss that this domain does not fit the latent structure, and that the items might be too severe for outpatients. While suicide ideation is a common feature in the depressed population, the CORE-OM items about self-harm and harm to others seem to generate very few affirmative responses. This was also seen in our analysis, visualized by the imbalance between persons and items at the right end of the graph in Fig. 2. Likely, the risk items are more relevant in inpatient psychiatric populations [24]. It is also possible that for our sample, representing a more diverse cultural background than the population used in the previous Swedish validation study [25], responses to the risk items could have been influenced by different cultural norms and beliefs about suicidal ideations.

In clinical practice with depressed and anxious patients, checking for risks is important to ensure that suicide thoughts are not overlooked, which is why we kept one risk item in the modified scale. An alternative could be to separate the risk domain from the scale, which has been discussed in previous CORE studies [10, 15, 24]. Additional ways can be considered to assess suicidality in the clinical encounter. While there is no golden standard, there are specific scales or suicide items that can be used in conjunction with the patient narrative [41].

The many high residual correlations (Table 3) between items likely affected model fit, due to local dependency [37]. This is also related to the dimensionality of the scale, since dependent items might breach the assumption of unidimensionality [42]. The high residual correlations in part confirmed the four conceptual domains in CORE-OM. The relation between the four domains has, however, not been clearly established in previous studies, which makes assumptions about the dimensionality of the scale difficult. We chose a unidimensional Rasch model for the analysis based on the CORE-OM assumption of a common higher ordered construct [43] of psychological distress. Although, a multidimensional Rasch model [44] might have provided a different outcome and therefore should be considered in future research on the CORE-OM and measures of psychological distress.

Like previous studies [15, 24], our analysis did not support a structure of four psychometrically separate domains. While previous research mainly suggests a latent structure of three dimensions [15]—negative, positive and risk items—the emotional component of the CORE-6D showed to be a unidimensional construct of psychological distress for our data. In the CORE-6D, there are no items from the wellbeing domain of the CORE-OM. Like previous research [24], our findings suggest that wellbeing and psychological distress are preferably measured as two different constructs. Other specific wellbeing scales could be an alternative in clinical practice when wellbeing is of primary concern to measure.

Our results showing that 69% of the patients improved significantly is a somewhat higher estimate than another Swedish study [45], presenting that 37% of patients receiving psychological treatment in primary care improved reliably on their CORE-OM score. Since we lacked repeated measures data for most of the sample, and only 40 participants had data for all three timepoints, we cannot draw conclusions from these sensitivity analyses. However, we wanted to include the findings since very few studies applying Rasch methodology on the CORE-OM have reported any data on sensitivity to change. It should also be noted that the definition of a meaningful change and/or clinically important difference is a common problem in sensitivity to change analyses [46, 47]. While most patients in our study would have ended their treatment when treatments goals were achieved, with improved health and functioning, patients in clinical practice may end their treatment for other reasons, for example a referral to another clinic, moving, dissatisfaction or inability to continue treatment. This warrants further studies to provide guidance on what is a minimal clinically important difference in person measures assessed with CORE-OM – which should go beyond statistical methods only and needs guidance from clinicians [47, 48] – and to evaluate change in psychological distress together with other linked person attributes.

While the emotional component of the CORE-6D showed the best fit for our data when we explored different item combinations, an important consideration was to include items from the trauma domain, since post-traumatic stress was common in the sample, and to increase PSI for individual measurement. While the suggested 8 item set improved PSI, one item (number 5) displayed DIF for age. This item had not displayed DIF in the previous analysis and to not risk decreasing the PSI, we decided to keep this item. Also, since most participants in this population were young (< 30 years) the dichotomization into equal groups for the analysis was not ideal to explore and conclude about DIF for age. This aspect remains to be investigated in a more representative sample in terms of age, and possibly adjustments could be made in post-statistical estimation, such as splitting data for this item.

The combination of 8 items suggests a brief alternative measure of psychological distress in Swedish depressed and anxious out-patients in multicultural areas. However, since our analyses were based on item reduction with data from assessments of the full CORE-OM, the modified item set warrants further psychometric investigation and validity testing. Likewise, the analyses of the shorter versions (CORE-10/CORE-6D) were also derived from the CORE-OM data, which means that the participants did not fill in the shorter versions separately. Caution must be taken when interpreting the results for these item sets, and generalizability cannot be assumed. Future validity studies of the CORE-10 and CORE-6D in clinical settings, using modern test theory, should be a welcome contribution.

### Study limitations

The most important limitation concerns the study sample, and how the naturalistic setting and procedures may have hampered the results. As described in the methods, patients at the clinic routinely filled in the CORE-OM assessment at their first visit, but for the other timepoints, routines were less structured, and many patients finished treatment without a follow up assessment. Detailed reasons for missing data were not known to the researchers or retrievable from the clinic. However, the primary aim was to check the fundamental properties and internal validity of the CORE-OM, for which one data collection point (i.e. the baseline data of n = 337) is sufficient.

The Swedish CORE-OM was translated according to rigorous standard procedures, with face validity explored in expert panels and in student and clinical populations. In these samples, with native Swedish speakers, the translation and validation worked well [25]. However, our sample was a convenience sample from a clinic in a multicultural area where many patients are not native Swedish speakers. Unfortunately, we did not have data on the participants’ native languages which could have enabled another person factor to check for DIF. We suggest that the phrasing of some items might be difficult to understand if language skills are limited. For example, items 6, 14, 23, 29 and 33 use Swedish words where a choice of simpler wording could be considered. Moreover, alternating between positive and negative items in a scale may be more difficult for non-native speakers. Additionally, contemporary anthropologists have argued that all distress can be viewed as ‘culture bound’ [49]. For example, the way people think of depression may be influenced by the cultural view of the individual and the role of the individual in society [50]. In our multicultural sample it cannot be ruled out that some of the validity problems were due to different understandings of the Swedish expressions. Since the number of migrants in Sweden has increased in recent years, and around 20% of the population has another native language, the issue of simple and concise items is essential. On the other hand, caution should be taken to changing the wording of items, since that may alter the linguistic nuance of the original items and possibly lose the intended meaning. To increase knowledge of how the items are perceived and understood in patients who are not native speakers, qualitative studies are warranted, which we suggest for future research.

Another limitation was the naturalistic data collection, where the CORE-OM data were collected and recorded as routine practice at the psychiatric clinic. Additional participant characteristics such as sociodemographic background and diagnosis for the whole sample would have been useful for the analysis and the interpretation of results. To minimize the risk of bias in the data collection, one of the authors (JGH) checked and transferred all data from the pen and paper format. For future development, electronic versions of the scale, sent to the patient before the appointment with data automatically recorded, could minimize data management bias. Such procedure would likely be less time consuming and give more time to focus on the patient narrative in line with a person-centered approach.

Our sample size (n = 337) was adequately powered according to recommendations (> 250 subjects). However, since some researchers suggest around n = 500 for polytomous scales, we pooled data from the repeated measures to obtain 521 observations. This would provide more robust calculations and enabled sensitivity analyzes of item stability over time. However, it has been suggested that as sample size increases, the number of items showing misfit will also increase [51]. In most situations, this type 1 error will occur in samples > 1000, but we cannot rule out that the repeated measure strategy affected some individual item misfit. To ensure that type 1 error did not bias the scale fit, we also checked the data from the first timepoint separately (n = 337) where we found similar misfit for the whole scale.

## Conclusion

Measurement properties for the Swedish CORE-OM showed high internal consistency and adequate targeting in psychiatric out-patients with depression and anxiety in a multicultural area. Despite the high reliability, several items, especially the risk items, deviated from the expected model. This indicates that the full version of the scale may not be a good match to the population. The shorter CORE-6D showed acceptable model fit but low reliability for individual measurement. Adding three items to include depressive and trauma-related content, resulted in a unidimensional item set with acceptable reliability, model fit and targeting, which could be an alternative brief measure of psychological distress in Swedish psychiatric out-patients in multicultural areas. Qualitative studies exploring the CORE-OM items in non-native speakers are needed.

## Data Availability

The data that support the findings of this study are available on request from the corresponding author, LD. The data are not publicly available due to ethical and legal restrictions to ensure that the privacy of research participants is not compromised.

## Abbreviations

CORE-OM: Clinical outcomes in routine evaluation—outcome measures
PROM: Patient reported outcome measures
CTT: Classical test theory
IRT: Item response theory
DIF: Differential item functioning
ITT: Intention-to-treat
PP: per protocol
